# MRI-based structural development of the human newborn hypothalamus

**DOI:** 10.1016/j.dcn.2026.101697

**Published:** 2026-02-14

**Authors:** Elizabeth Yen, Josepheen De Asis-Cruz, Jerod M. Rasmussen

**Affiliations:** a Tufts University School of Medicine, Boston, MA, USA; b Woman, Mother + Baby Research Institute, Tufts Medical Center, Boston, MA, USA; c Tufts Medicine Pediatrics-Boston Children’s, Boston, MA, USA; d Developing Brain Institute, Children’s National Hospital, Washington, DC, USA; e Department of Pediatrics, George Washington University School of Medicine and Health Sciences, Washington, DC, USA; f Department of Pediatrics, University of California, Irvine, CA, USA; g Department of Biomedical Engineering, University of California, Irvine, CA, USA; h Department of Anatomy and Neurobiology, University of California, Irvine, CA, USA

**Keywords:** Hypothalamus, Infant, MRI, Sex, Smoking, Prenatal

## Abstract

**Background:**

Preclinical evidence suggests that intrauterine exposures can impact hypothalamic structure at birth and future disease risk, yet early human data are limited. Because the hypothalamus regulates critical early life processes including sleep, growth, stress regulation, and metabolic control, characterizing its structural maturation and how it relates to developmental conditions and exposures is essential for understanding links to later health.

**Methods:**

We measured hypothalamic volumes from T1-weighted MRI scans in the dHCP study (631 newborns; 699 observations). To characterize normative development, we examined associations with postmenstrual age (PMA, sum of gestational and chronological age) at scan and sex. Given the wide range of gestational age (GA) at birth in this sample, we also tested GA as a key intrauterine influence. In addition, we evaluated cigarette smoking during pregnancy, a perinatal risk factor with biological plausibility (*e.g.,* effects on growth and neuroendocrine function). Finally, we tested whether these associations persisted in an independent adolescent cohort from the ABCD study (11,207 adolescents; 16,934 observations).

**Results:**

Absolute hypothalamus volume increased with PMA (+5.5 %/week, t = 39.9, p < 10⁻¹⁰), but not after adjusting for brain volume (t = 1.2, p = 0.24). Males showed larger absolute (+3.3 %, t = 3.2, p = 0.002) but smaller relative volumes (t = -2.8, p = 0.005). Lower GA was linked to larger relative volume (t = -6.5, p < 10⁻⁹), with sex moderation (t = -2.4, p = 0.019). Smoking during pregnancy was associated with smaller newborn hypothalamus volume (t = -2.05, p = 0.04; dose dependence t = -2.4, p = 0.03). In adolescents, maternal smoking, but not categorical preterm birth, was linked to reduced hypothalamus volume (t = -2.8, p = 0.005).

**Conclusions:**

Early-life hypothalamic volume reflects both normative growth and vulnerability to intrauterine exposures, with smoking-related differences persisting into adolescence.

## Highlights

• Study considers the hypothalamus as an underexplored target of prenatal exposures.
• GA, PMA, sex, and smoking associated with hypothalamic volume in newborns.
• Limited evidence that effects of GA may be transient.
• Effects of smoking during pregnancy may last into adolescence.

## 1 Introduction

Advances in brain magnetic resonance imaging (MRI) continue to enhance our understanding of the developing fetal and newborn brain (Andescavage et al., 2017, Bethlehem et al., 2022, Dubois et al., 2021, Lautarescu et al., 2024, De Asis-Cruz et al., 2020a). More specifically, these tools are beginning to uncover how pregnancy-related conditions affect brain development and long-term health outcomes, including physical growth, cognitive function, school performance, and psychiatric illness (Chilton et al., 2017, Dabelea and Crume, 2011, Ward et al., 2013, Wu et al., 2020). Much of the attention has focused on global brain measures and cortical and subcortical systems that support higher-order functions (Yen et al., 2023). Whereas considerably less focus has been directed to the hypothalamus, a “small but mighty” brain structure responsible for physiological homeostasis and control of survival-oriented behaviors (Maroso and Stern, 2023).

As a highly conserved structure across vertebrate species (Chen et al., 2025, Chen et al., 2022), the hypothalamus organizes fundamental functions, including thermoregulation, wake-sleep cycles, energy metabolism, feeding and suckling, drinking, and reproduction (Saper and Lowell, 2014). It is among the earliest regions of the central nervous system to develop (Placzek et al., 2025), as early as the third week of gestation in the form of the neural plate (Langman’s, 2012). This early window of development renders the hypothalamus especially sensitive to the effects of in-utero exposures and pro-inflammatory conditions (*e.g.,* cigarette smoking), which are likely to disrupt energy homeostasis and result in early behavioral changes predisposing offspring to adult-onset diseases (Brett et al., 2015, Nunez-Rios et al., 2023, Pimentel et al., 2014, Rasmussen et al., 2022, Sewaybricker et al., 2024, Ullah et al., 2023). Despite the importance of understanding early-life human hypothalamic development, imaging research on this topic has been hindered by its small size (Ruzok et al., 2022) and the lack of infant-specific tools available for its delineation, with a few exceptions (Diéguez et al., 2022, Pollatou et al., 2024).

To address this gap, we leveraged an infant-optimized, registration-based pipeline that enables automated FreeSurfer-compliant segmentation of the neonatal hypothalamus and its subunits, validated across > 200 neonatal MRIs from four Environmental Influences on Child Health Outcomes (ECHO) sites (Rasmussen et al., 2024). Using this tool, our goals were threefold. First, we characterized normative hypothalamic growth in relation to postmenstrual age (PMA) at scan and sex in a publicly available large newborn dataset (Developing Human Connectome Project, dHCP (Fitzgibbon et al., 2020)). Second, given the wide range of gestational age (GA) at birth in the dHCP dataset, we examined GA as a key intrauterine condition influencing hypothalamic development. Third, we evaluated maternal cigarette smoking during pregnancy. Smoking is a leading preventable cause of pregnancy complications, affecting ∼5 % of pregnancies (Kipling et al., 2024) in the United States. It is strongly patterned by sociodemographic factors such as age, income, education, and employment, and its dose-dependent effects on fetal growth and infant outcomes are well established (Garrett et al., 2019, Sequi-Canet et al., 2022, Sun et al., 2023). Biologically, smoking exerts pro-inflammatory effects, disrupts hypothalamic–pituitary–adrenal (HPA) pathways, and alters feeding and sleep regulation (Chen et al., 2012, Chen et al., 2024, Rohleder and Kirschbaum, 2006), providing a mechanistic rationale for its potential influence on hypothalamic development. To test whether associations observed in newborns persisted after postnatal exposures, we examined an independent adolescent cohort from the Adolescent Brain and Child Development (ABCD) study (Karcher and Barch, 2021). Finally, given the sexual dimorphism in brain development (McCarthy, 2020, Wheelock et al., 2019) and the direct role of the hypothalamus via the hypothalamic–pituitary–gonadal (HPG) axis, we also explored sex differences in hypothalamic development.

## 2 Methods

### 2.1 Data collection: dHCP sample

**Fig. 1:**
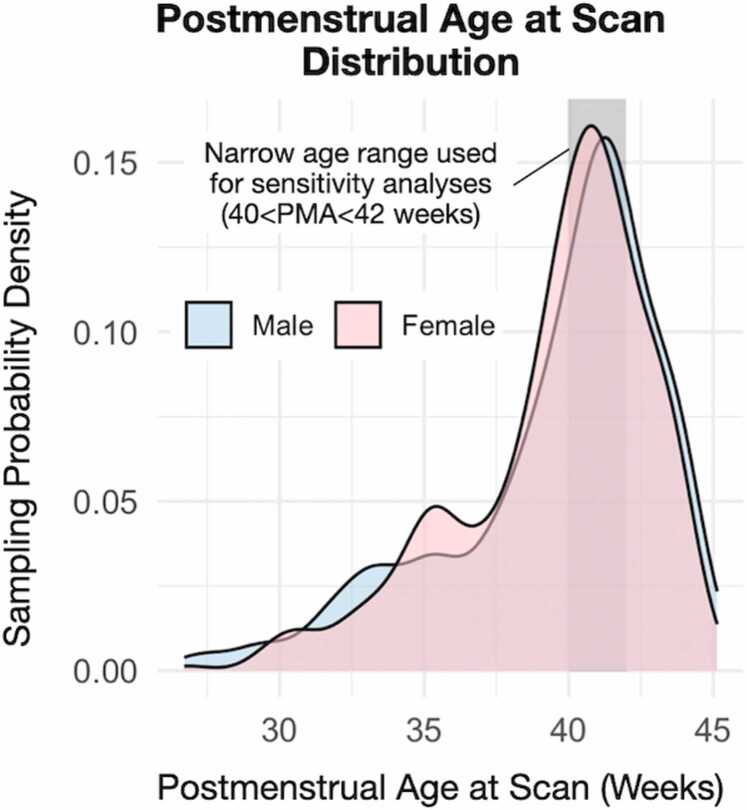
Postmenstrual age at scan distribution by sex. No significant sex differences were observed in PMA at scan, GA, or maternal smoking status.

To study the characterization of typical development, MRIs of healthy term-born and preterm infants were obtained from the dHCP study, located in the National Institute of Mental Health Data Archive (Collection #3955). Additional details about this cohort are well described elsewhere (Karolis et al., 2025, Makropoulos et al., 2018). For the current study, 699 observations of 631 unique participants were analyzed. Infants born preterm were included to increase variation in postmenstrual age (PMA) at scan, thereby improving the precision of estimated age-related slopes. Demographic and exposure characteristics are described in Table 1. Pregnant women were enrolled from mid to late pregnancy, and imaging occurred between 26.7- and 45.1-weeks PMA (Fig. 1). Exclusion criteria were contraindication to MRI, inability to tolerate scanning (e.g., thermoregulation issues, sepsis, need for invasive mechanical ventilation), and language barriers impacting the process. Data collection and dissemination were approved by the United Kingdom Health Research Authority (Research Ethics Committee reference number: 14/LO/1169). In addition, a partial replication analysis was used to test the persistence of effects into adolescence using the ABCD Study as approved by a central Institutional Review Board. For a more complete description of recruitment and inclusion criteria, we refer the reader to articles by (Karcher and Barch, 2021, Saragosa-Harris et al., 2022). Written parental consent was obtained in all cases.

**Table 1:** Study demographics. The newborn participants were split 53.6 % female/46.4 % male. Maternal smoking during pregnancy was recorded in 3.3 % of participants. *Males were ∼75 g larger in birth weight than their female counterparts after adjustment for GA (p = 10^−4^). No other sex differences in table demographics were observed (all p > 0.4) except for brain and hypothalamus volume (discussed in detail below).

**Characteristics**	**Mean**	**Range**	**IQR**
Postmenstrual Age at Scan (weeks)	39.6	26.7–45.1	38.1/40.4/42.0
Gestational Age at Birth (weeks)	36.8	23.0–42.3	34.0/38.9/40.4
Postnatal Age at Scan (weeks)	0.4	0.0–2.8	0.1/0.2/0.5
Birth Weight (kg)*	2.8	0.5–4.8	2.1/3.1/3.6
Birth Length (cm)	50.7	20–75	49/51/53.5
Brain Volume (cm³)	408	111–636	357/418/468
Hypothalamus Volume (mm³)	285	82.8–486	252/291/326

### 2.2 Data collection: dHCP imaging

T1-weighted images were used to segment the hypothalamus (see *Infant Hypothalamus Segmentation* below). For the dHCP study, MRI was performed at the Evelina London Children’s Hospital on a 3 T Philips Achieva system with a dedicated 32-channel neonatal head coil. T1-weighted inversion recovery images were collected as part of a newborn imaging battery (TR= 4795 ms; TI= 1740 ms; TE= 8.7 ms; SENSE factor: axial= 2.26, sagittal= 2.66). One image stack each (5m45s) in sagittal and axial slice orientation was acquired with 0.8 mm in-plane resolution and 1.6 mm slices overlapped by 0.8 mm. Images were reconstructed at a 0.8 mm isotropic resolution and downloaded for use here as provided by the dHCP data repository.

### 2.3 Infant hypothalamus segmentation

Infant hypothalamic volumes were derived using an adapted version of the recently published and publicly available (https://github.com/jerodras/neonate_hypothalamus_seg) segmentation pipeline, segATLAS (Rasmussen et al., 2024). In brief, this method uses a FreeSurfer-compatible hypothalamus atlas in high-quality PMA-appropriate template space (Baby Connectome Project, BCP) (Howell et al., 2019) and robust infant brain extraction methods (ANTsPyNet, https://github.com/ANTsX/ANTsPyNet) consistent with the BCP template definitions. However, given the high-quality image registrations publicly shared by dHCP and their now ubiquitous community use, we integrated the existing dHCP registrations to the 40-week Serag template into the existing segATLAS pipeline. This was done by creating an atlas in the 40-week Serag template space using ANTs non-linear registration. Estimates of volume were then based on a soft-segmentation approach, adding up fully- and partially-volumed voxels. Similarly, brain volume (BV) is based on the dHCP-provided brain masks and used here to reflect global brain size, controlling for non-specific hypothalamic growth. In addition, to test the specificity of effects (*i.e.,* not generalizable to other subcortical structures), bilaterally averaged amygdala volumes were chosen as a negative control (Sewaybricker et al., 2024) based on their comparable size to the hypothalamus and relative spatial proximity as a central brain structure.

### 2.4 Modeling effects of growth and prenatal predictors

As a first step towards understanding hypothalamic growth in early life, we constructed a base model using linear mixed-effects regression to regress hypothalamus volume onto PMA at scan and sex, and residual diagnostics were used to flag outliers (>3 standard deviations, n = 7) and used in sensitivity analyses as quality control (QC). This base model did not include other prenatal or demographic predictors. Further, to understand hypothalamic growth beyond global increases in brain size, we used two complementary approaches. First, BV was added as a term to the base linear model, allowing for independent effects (i.e., growth of the hypothalamus) beyond global size. Alternatively, hypothalamus volume was expressed as a ratio relative to BV, thereby forcing a relationship between hypothalamus volume and BV and accounting for non-linearity (a ratio approach). The rationale for these two approaches is that while the ratio approach accounts for non-linearities in growth that are not accounted for in linear regression, it does so by forcing BV variance into the outcome. Because growth in this age range is expected to be approximately linear, the non-ratio approach was our first choice while confirming that any observed relationships also hold true using the ratio approach as described in the Supplementary Material.

GA and maternal cigarette smoking during pregnancy were included as prenatal predictors of early life hypothalamus volume over and above adjustment for PMA, BV, and sex (base model). GA was estimated from the last menstrual period and/or confirmed by early ultrasound. PMA was defined as GA plus chronological age (i.e., the time since birth). Maternal smoking status was collected by survey at the time of enrollment and was coded as a dichotomous yes/no answer based on self-report. Additionally, for a dose-response analysis, we used the numeric response to “Average number of cigarettes smoked a day at that time.” Of note, one observation was winsorized to two standard deviations from the mean to reduce the influence of this single data point. A full linear mixed-effects model was constructed by expanding the base model above to include prenatal predictors. Final model selection was performed using a best subset analysis based on adjusted R-squared selection criteria implemented using the “leap” package in R version 4.3.3. Lastly, with the exception of smoking exposure in the dHCP sample (omitted due to sample size), sex-specific effects were considered by testing for sex interactions.

### 2.5 Partial replication analysis in an independent adolescent cohort

To determine if neonatal and maternal factors have a persistent effect on hypothalamic volumes extending into adolescence, we conducted a partial replication analysis assessing the effects of chronological age, sex, GA, and maternal smoking during pregnancy on hypothalamus volume in an independent cohort (Casey et al., 2018, Garavan et al., 2018). High-quality minimally preprocessed (Hagler et al., 2019) T1-weighted images from 11,207 individuals (16,934 observations, 9380 unique families) in the ABCD study were considered for analysis (ABCD Data Release 5.1, DOI: 10.15154/z563-zd24). Imaging data were available at baseline (∼10 years of age) and two-year follow-up. Hypothalamus volume was derived using FreeSurfer’s *mri_segment_hypothalamic_subunits*(Billot et al., 2020) tool and harmonized by accounting for site effects using ComBat batch correction (Orlhac et al., 2022). Age at interview (interview_age), sex (demo_sex_v2), and brain volume (mrisdp_604) were based on ABCD-provided tabulated measures. The rationale for using ABCD-provided tabulated measures of brain volume is that these measures are the consortium-verified standard, ensuring more rigorous quality control and maximum comparability across the broader ABCD literature. Information on the exact GA was not available, therefore, preterm birth status (devhx_12a_p, yes/no < 37-weeks GA) was used as a proxy. Maternal smoking status during pregnancy was available for analysis (devhx_9_tobacco, “Once you knew you were pregnant, were you using any of the following: Tobacco?”) and chosen as a harmonized variable with respect to the dHCP dataset. Responses of “Don’t know” were (conservatively) recoded as “No”, and sensitivity analyses excluding them were conducted to assess the impact of that decision on the model. As above, a parsimonious linear mixed-effects model considering chronological (adolescent) age, sex, and BV was used to test for an association with hypothalamus volume and nesting for participant and family structure using random intercepts. Informed by the dHCP findings, this model was repeated testing for an age-by-sex interaction effect. A second linear mixed-effects model, adding maternal smoking status during pregnancy and preterm birth status, was used to test for an association with hypothalamus volume over and above age, sex, and brain volume. As the primary model testing for a partial replication of the effects of GA and maternal smoking during pregnancy, this model was repeated censoring for images flagged as low quality by ABCD using *iqc_t1_1_qc_score* (n = 178 total observations censored). Lastly, a full mixed-effects model, adding potentially confounding factors (area deprivation index: adi_weighted; parent education: prnt_educ_max; household income: prnt_comb_income_cont; puberty status: ppdms_score; race: race_cleaned; and ethnicity: demo_ethn_v2; 14,446 complete observations across 7732 individuals), was used to test for an independent association with hypothalamus volume.

## 3 Results

### 3.1 Postmenstrual age at scan is associated with absolute, but not relative hypothalamus volume

**Fig. 2:**
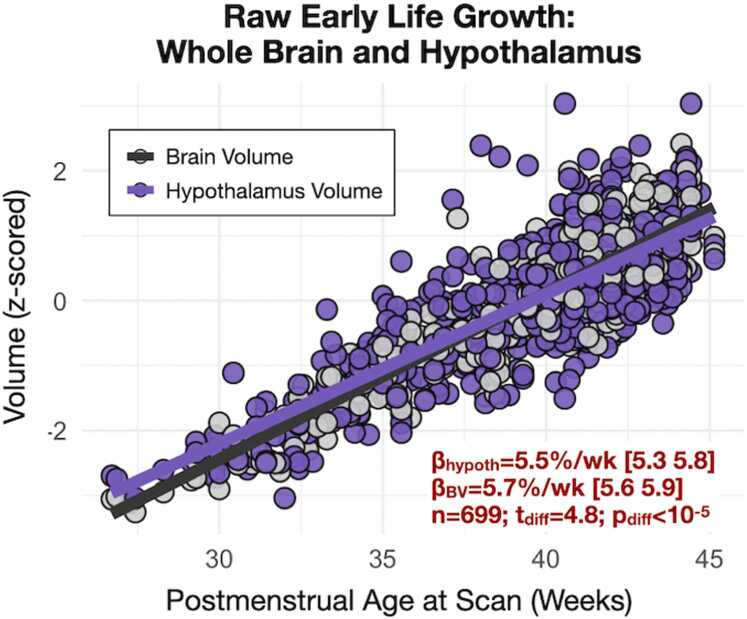
Postmenstrual age at scan is associated with raw (unadjusted) hypothalamus volume. Hypothalamus volume was associated with PMA. While the slope of association with age differs between *absolute* hypothalamus volume and BV (p < 10^−5^), no association was observed when expressing hypothalamus volume as a measure *relative* to BV.

Raw, unadjusted (absolute) hypothalamus volume was strongly associated with PMA (Fig. 2, +5.5 % increase in volume per week, t = 39.9, p < 10^−10^, 699 observations across 631 unique participants). The normalized slope of the association with PMA is significantly different when compared to that of BV (t = -4.8, p < 10^−5^), suggesting that the hypothalamus grows at a slower rate relative to BV. Evaluation of the amygdala (similar in size to the hypothalamus) also showed a significantly slower growth rate when compared to BV (t = -3.2, p < 0.001). There were no significant differences when comparing the relative hypothalamus to amygdala growth (t = 1.5, p = 0.12). This suggests that the observed difference in hypothalamus slope is not specific to the hypothalamus, but rather generalizable to at least one other subcortical structure. In addition, hypothalamus volume was not significantly associated with PMA when adjusting for BV in a linear model (t = 1.2, p = 0.24) or as a ratio (t = -1.0, p = 0.32). Hence, while the slope of association with PMA differs between *absolute* hypothalamus volume and BV, these differences were not observed when expressed as *relative* measures.

### 3.2 Infant sex is associated with absolute and relative hypothalamus volume

In addition to the significant effect of PMA, infant sex also significantly affects BV and hypothalamus volume. Compared to females, males had larger *unadjusted* global brain and hypothalamus volumes (BV: male=+7.0 %, t = 8.9, p < 10^−10^, hypothalamus: male=+3.3 %, t = 3.2, p = 0.002). A significant effect of sex on hypothalamus volume remained in both the adjusted model and the ratio model, with smaller *relative* hypothalamic measures in males compared to females (t = -2.8, p_adj_=0.005; t = -3.7, p_ratio_<10^−5^). This effect was not observed using amygdala volumes (t = 0.3, p_ratio_=0.76), suggesting that the observed effects of sex were specific to the hypothalamus.

### 3.3 Gestational age at birth and maternal smoking during pregnancy are associated with infant hypothalamus volume

**Fig. 3:**
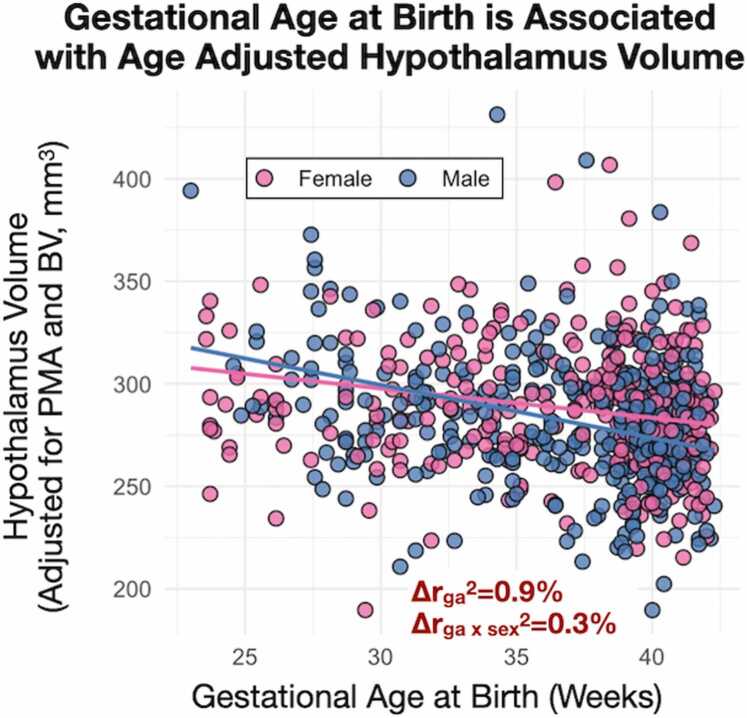
Gestational age and early life hypothalamus volume. a) GA was negatively associated with hypothalamus measures adjusted for PMA and BV (t = -6.5; p < 10^−9^) and exhibited sex-specific associations (t = -2.4; β_m,brain_>β_f,brain_: p = 0.019).

**Fig. 4:**
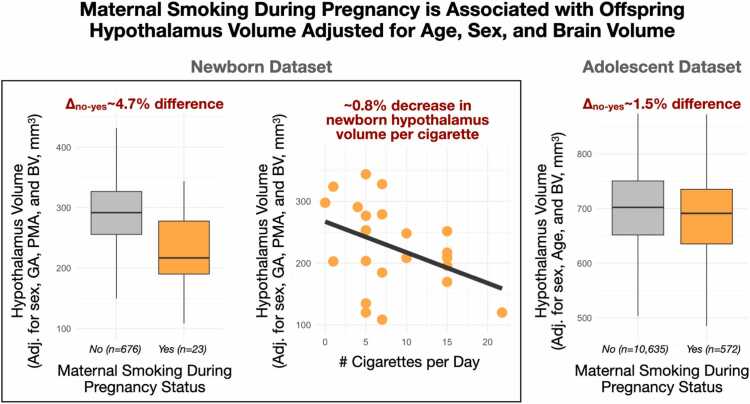
Effect of maternal cigarette smoking status on offspring hypothalamus volume. Offspring born to mothers who smoked during pregnancy exhibited smaller postnatal hypothalamus volume (left panel). This effect was dose-dependent (central panel) and partially replicated in an independent adolescent cohort (right panel).

GA and maternal smoking during pregnancy were associated with infant hypothalamus volume after adjusting for PMA, BV, and sex (Fig. 3, Fig. 4**,** Table 2). The direction of association with GA was negative, suggesting that early birth is consistent with an enlarged hypothalamus relative to BV (Fig. 3). Notably, this direction was consistent with a sensitivity analysis using preterm birth status (N = 699, t_term_= -5.3, p_adj_<10^−6^) as a coarse proxy for GA (and thereby reducing collinearity). To further address potential confounding, we analyzed a cross-sectional subset limited to PMA> 38 weeks (n = 523). In this subset, PMA did not significantly differ between preterm and term froups (t_term_= 0.2, p_adj_=0.86), yet preterm birth status remained a significant predictor of hypothalamic volume (n = 523, t_term_= -6.2, p_adj_<10^−6^). We observed sex-specific effects with point estimates suggesting males have larger adjusted relative hypothalamus volumes early in life (∼25 weeks GA) but with a larger negative GA slope compared to females. Maternal smoking (dichotomous yes/no) during pregnancy was associated with a smaller hypothalamus. Notably, we also observed a dose-dependent effect in the subset of mothers (n = 23) who reported smoking during pregnancy (t = -2.4, p = 0.03) (Fig. 4). This effect was not observed when using amygdala volume as a comparison (t_yes/no,amyg_=0.23, p_yes/no,amyg_=0.82; t_dose,amyg_=-1.2, p_dose,amyg_=0.26), and sensitivity analyses removing QC flagged observations did not qualitatively change the model (all effect sizes remained within 1 % of their uncensored value and all p < 0.05). Model diagnostics for multi-collinearity suggested that while the effects of PMA and BV are potentially inflated in the model, effects of sex, GA and smoking are not (VIF_PMA_= 6.6, VIF_BV_= 5.9, VIF_GA_= 1.7, VIF_sex_= 1.1, VIF_smoking_= 1.0).

**Table 2:** Linear mixed-effects model predicting whole hypothalamus volume *(N = 699 observations from 631 participants).*

**Variable**	**Estimate (95 % CI)**	**Std. Error**	**df**	**t Value**	***P*****Value**
Intercept	-6.01 (-45.38, 33.37)	20.08	676.2	-0.30	0.76
Brain Volume (mm^3^)	0.00056 (0.00050, 0.00062)	0.00003	630.4	19.91	**< 0.001**
Postmenstrual Age (weeks)	2.93 (1.30, 4.55)	0.83	685.9	3.52	**< 0.001**
Gest Age Birth (weeks)	-2.05 (-2.67, −1.43)	0.31	588.0	-6.52	**< 0.001**
Mother Smoking (Yes)	-13.33 (-26.12, −0.54)	6.51	495.8	-2.05	**0.04**
Male Sex	-6.11 (-10.87, −1.35)	2.43	548.5	-2.52	**0.01**

The associations between infant hypothalamus volume and GA, sex, and maternal smoking survived (and often improved in effect size and significance) exhaustive testing in the context of different models (ratio vs. non-ratio), global adjustment covariates (brain volume, bilateral amygdala volume), and subsets of data with more narrow limits (PMA 40–43 weeks, GA 39–41 weeks, near full-term >36 weeks). Such exhaustive testing was used to minimize the confounding between GA and PMA at scan present in the dHCP dataset (see Supplementary Materials S1*: Multiverse Analyses*).

### 3.4 Maternal smoking during pregnancy is associated with adolescent hypothalamus volume

Effects of adolescent age, sex, preterm birth status, and maternal smoking during pregnancy on hypothalamus volume were considered in the ABCD sample. Adolescent age was associated with hypothalamus volume in both absolute and adjusted measures (t_age,abs_= 37.6, p_age,abs_<10^−10^; t_age,adj_= 39.9, p_age,adj_<10^−10^) in a direction supportive of protracted growth during the adolescent period, including over and above global volume. Absolute and relative measures of hypothalamus volume were associated with sex (males 1.1 % larger on average; t_sex,abs_= -10.2, p_sex,abs_<10^−10^; t_sex,adj_= -2.3, p_sex,adj_= 0.02), and sex-specific associations with age were significant in both absolute and relative terms (t_age-sex,abs_= -5.6, p_age-sex,abs_<10^−7^; t_age-sex,rel_= -5.3, p_age-sex,rel_<10^−6^). Collectively, these findings suggest that the hypothalamus during early adolescence is characterized by sex-specific growth trajectories in which the male hypothalamus volume is larger than in females and increasing in magnitude. There was no observed main (t = -0.9, p = 0.39) or sex-specific (t = 0.7, p = 0.47) association with preterm birth status. However, there was a significant main effect of maternal smoking during pregnancy (t_smoking_= -3.0, p_smoking_=0.002) that persisted when censoring for low data quality (t_smoking,censored_= -3.1, p_smoking,censored_= 0.002) and survived adjustment for potentially confounding variables (t_smoking_= -2.8, p_smoking_= 0.005). In addition, sensitivity analyses excluding participants with a “Don’t know” for maternal smoking status did not meaningfully affect the model (t_smoking_= -3.0, p_smoking_= 0.003). Consistent with observations in the dHCP dataset, our result suggested a similar reduction in adolescent hypothalamus volume (-1.5 % on average) in the presence of maternal smoking during pregnancy (Fig. 4). There was no significant interaction effect between maternal smoking during pregnancy and offspring sex (t = 0.6, p = 0.54).

## 4 Discussion

Using large, well-characterized neuroimaging datasets, we demonstrate that early-life hypothalamus volume is associated with normative development (PMA, sex), as well as with pre-/perinatal conditions and exposures (GA and maternal smoking during pregnancy). These findings are further expanded by four key observations. First, although raw, unadjusted hypothalamus volume was positively associated with PMA, this association was no longer present when controlling for global BV, either through covariate adjustment or ratio scaling. Second, GA was negatively associated with adjusted hypothalamus volume; however, evidence from adolescence suggests that any lasting impact of preterm birth on hypothalamus volume may be subtle or undetectable as observed here. Third, our findings suggested sexual dimorphism in absolute and relative hypothalamus volume and evidence of sex-specific effects on the associations between volume and age measures across development. Finally, maternal smoking during pregnancy was linked to smaller hypothalamus volume at birth, and this association was partially replicated in a large-scale, nationally representative adolescent sample in both magnitude and direction of effect.

Unadjusted analyses from the dHCP newborn dataset revealed a linear increase in hypothalamus and brain volumes with PMA, with BV having a steeper slope. However, this association was no longer present when adjusting for BV, providing mixed evidence that hypothalamic growth during this early life period may reflect global (or local) expansion. In adolescence, however, hypothalamus volume was positively associated with age in both absolute and BV-adjusted terms. Notably, these observations are consistent with prior literature (Isıklar et al., 2022) in smaller samples (n = 26, 0–6 mos; n = 43, 10–12 yrs) demonstrating no significant increase in hypothalamus volume relative to BV in the early life period, yet a significant increase in hypothalamus volume relative to BV in the adolescent period. Together, these data support a model in which hypothalamic growth broadly parallels global brain development, yet exhibits distinct, phase-specific acceleration patterns likely reflecting the structure’s responsiveness to age-related physiological changes such as puberty.

We observed that lower GA at birth was associated with larger hypothalamus volume after adjusting for PMA and BV. This relationship may reflect early adaptation or accelerated hypothalamic development in response to the extrauterine environment. One possible explanation is that preterm birth acts as a developmental catalyst, triggering early physiological transitions, including hormonal, metabolic, and neurodevelopmental changes that transiently accelerate hypothalamic growth, analogous to increased functional connectivity in stress-related circuits in premature infants (De Asis-Cruz et al., 2020b). Interestingly, prior work suggests that hypothalamic *subunit* growth in preterm infants may diverge early, potentially contributing to smaller volumes observed in adults born preterm (Ruzok et al., 2022). However, it should be noted that the evidence presented here suggests that the impact of preterm birth on whole hypothalamus volume is no longer detectable during adolescence. Taken together, we speculate that the impact of preterm birth on hypothalamus structure persists across the lifespan but age-related and subunit-specific physiological changes, *e.g.,* puberty, may mask such impact during adolescence. Alternatively, our lack of an observation may simply be due to measurement uncertainty, as the ABCD dataset has notable limitations on the comprehensive study of the effects of preterm birth including: a categorical definition of pre-term birth that limits sensitivity to small and potentially non-linear effects, and exclusion criteria including GA < 28 weeks, birth weight < 1200 g, and birth complications resulting in hospitalization for more than 1 month (Palmer et al., 2021).

We identified multiple sex-specific effects in hypothalamic development, including both volumetric differences and distinct associations between hypothalamus volume and age. In newborns, we observed that the hypothalamus is larger in males than in females on average. Our finding is consistent with an abundance of literature supporting this premise (Kaczkurkin et al., 2019, Khan et al., 2024, Wierenga et al., 2014, Xu et al., 2025). However, when adjusting for global brain measures, the female hypothalamus was observed to be larger. Notably, this finding is consistent with recent literature demonstrating a similar effect in the newborn caudate nucleus using a large-scale multi-site secondary analysis (Alex et al., 2024), and newborn total gray matter volumes from the same cohort considered here (but with no measures for the hypothalamus available) (Khan et al., 2024). Such relative differences may stem from differential timing of regional brain growth as a function of age and sex, likely driven by complex physiological changes and needs (Goddings et al., 2014, Thomaz et al., 2024, Wagner et al., 2023, Zhu et al., 2024). In contrast, by adolescence, we found that the hypothalamus was larger in males than in females, both in absolute and relative terms. These findings motivate future research examining how developmental timing, hormonal regulation, and energetic needs influence hypothalamic growth trajectories.

In addition to sex-based differences in hypothalamus volume, we also observed sex-specific associations between volume and age. Specifically, relative to global brain development across GA, the hypothalamus grows at a slower rate, with males affected to a greater extent than females. Due to the absence of any functional or behavioral measurements, the implications of such slower relative growth are unclear at this point. However, the current study provides an opportunity to validate these findings further and add behavioral or functional effects to understand the clinical relevance of the relatively slower hypothalamic growth rate in males than females.

We observed reduced hypothalamus volume in both newborns and adolescents whose mothers reported smoking during pregnancy, with a dose-dependent relationship evident in newborns. Nicotine and other neuroactive compounds in cigarette smoke readily cross the placenta, exposing the rapidly developing fetal brain to harmful toxins (Jauniaux and Burton, 2007). Nicotine acts by binding to nicotinic acetylcholine receptors (nAChRs), which are critical for neurodevelopmental processes including neurogenesis, neuronal migration, and synaptogenesis (Dwyer et al., 2008, Matta et al., 2021). In utero exposure to nicotine can therefore interfere with neurotransmitter signaling and disrupt these fundamental cellular events (Dwyer et al., 2008, Ernst et al., 2001).

The observed reduction in hypothalamus volume, but not amygdala volume, supports the unique vulnerability of the hypothalamus during fetal and early postnatal development. While both structures express nAChRs and might be expected to show similar susceptibility, differential sensitivity may reflect variation in developmental trajectories (Kim et al., 2020, Mulc et al., 2024), critical periods for cellular events, the specific nAChR subtypes expressed (Sharp, 2019, Zoli et al., 2015), and the timing of prenatal exposure relative to these sensitive windows. Importantly, the presence of a dose-response relationship, persistence of effects into adolescence, and robustness to adjustment for postnatal sociodemographic factors together strengthen the inference that maternal smoking has a sustained impact on hypothalamic development.

We acknowledge that the main effect of maternal smoking exposure in our neonatal sample achieved a significance level that, unlike the effects of GA, would not survive stringent correction for multiple comparisons across the two exposure models (GA and smoking). However, we consider this result a discovery-based finding corroborated by a significant dose-response effect and a partial replication in the independent ABCD study. These converging results reduce the likelihood of a Type I error, though further dedicated studies are needed to confirm the precise effect size in neonatal populations.

Our study has several limitations. First, in the newborn sample, postmenstrual age (PMA) at scan and gestational age (GA) at birth are partially confounded, as dHCP scans were collected within three postnatal weeks on average. We addressed this with variance inflation factors and subgroup sensitivity analyses, but findings related to GA should still be interpreted with caution and attempted replication in future work. Second, we modeled the hypothalamus as a single structure. Given its heterogeneous nuclei and specialized functions, this approach oversimplifies its organization. Future efforts should aim to parcellate the newborn hypothalamus into more structurally and functionally informed subunits (Jensen et al., 2024). Third, although we considered cigarette smoking as an exposure due to its biological plausibility, this may oversimplify the complex effects of sociodemographic factors. Further analyzing these characteristics individually could help disentangle direct biological from indirect contextual influences. Finally, our analyses were largely cross-sectional, and the two cohorts we studied span a large age gap. While preclinical work strongly supports prenatal effects on hypothalamic development, longitudinal imaging will be essential. The forthcoming Healthy Brain and Child Development (HBCD) study (Nelson et al., 2024) offers a unique opportunity to test and extend these hypotheses by incorporating developmental trajectories, deeper socioeconomic characterization, and specific biological mechanisms. For example, future work should investigate maternal pre-pregnancy health status (e.g., body mass index) as a potential source of low-grade chronic inflammation that may impinge upon hypothalamic development alongside smoking.

The current study highlights the feasibility of studying the MRI-based structure of the developing hypothalamus and presents new insights into how perinatal factors may shape life course outcomes through their effects on this “small and mighty” brain region. Our study advances the premise that hypothalamic development is shaped by gestational age at birth, maternal smoking during pregnancy, and provides further evidence for sex-specific programming and developmental effects. Using two large developmental cohorts, our study provided robust evidence that the hypothalamus is a developmentally sensitive and underexplored target of perinatal influences. Given the hypothalamus’ central role in coordinating metabolic, endocrine, and behavioral systems vital for early survival and lifelong adaptation, understanding its development is essential for uncovering how early life exposures shape trajectories of health and disease.

## 5 Funding

10.13039/100000071NICHD R00 HD-100593 to JMR; 10.13039/100000025NIMH R01 MH-138481 to JMR

## CRediT authorship contribution statement

All authors contributed to the conceptualization of this project; JMR conducted analyses in consultation with EY and JDC; and all co-authors contributed to the writing process. All authors approved the submitted version. This manuscript has not been accepted or published elsewhere.

## CRediT authorship contribution statement

**Elizabeth Yen:** Writing – review & editing, Writing – original draft, Methodology, Conceptualization. **Josepheen DeAsis-Cruz:** Writing – review & editing, Writing – original draft, Methodology, Conceptualization. **Rasmussen Jerod:** Writing – review & editing, Writing – original draft, Visualization, Project administration, Methodology, Formal analysis, Conceptualization.

## Declaration of Competing Interest

The authors declare that they have no known competing financial interests or personal relationships that could have appeared to influence the work reported in this paper.

## Data Availability

Data will be made available on request.
